# Cold Physical Plasma in Cancer Therapy: Mechanisms, Signaling, and Immunity

**DOI:** 10.1155/2021/9916796

**Published:** 2021-12-24

**Authors:** Fatemeh Faramarzi, Parisa Zafari, Mina Alimohammadi, Mohammadreza Moonesi, Alireza Rafiei, Sander Bekeschus

**Affiliations:** ^1^Student Research Committee, School of Medicine, Mazandaran University of Medical Science, Iran; ^2^Department of Immunology, School of Medicine, Mazandaran University of Medical Science, Sari, Iran; ^3^Department of Immunology, School of Medicine, Shahid Beheshti University of Medical Science, Tehran, Iran; ^4^Department of Hematology, School of Medicine, Tabriz University of Medical Sciences, Tabriz, Iran; ^5^ZIK plasmatis, Leibniz Institute for Plasma Science and Technology (INP), Greifswald, Germany

## Abstract

Despite recent advances in therapy, cancer still is a devastating and life-threatening disease, motivating novel research lines in oncology. Cold physical plasma, a partially ionized gas, is a new modality in cancer research. Physical plasma produces various physicochemical factors, primarily reactive oxygen and nitrogen species (ROS/RNS), causing cancer cell death when supplied at supraphysiological concentrations. This review outlines the biomedical consequences of plasma treatment in experimental cancer therapy, including cell death modalities. It also summarizes current knowledge on intracellular signaling pathways triggered by plasma treatment to induce cancer cell death. Besides the inactivation of tumor cells, an equally important aspect is the inflammatory context in which cell death occurs to suppress or promote the responses of immune cells. This is mainly governed by the release of damage-associated molecular patterns (DAMPs) to provoke immunogenic cancer cell death (ICD) that, in turn, activates cells of the innate immune system to promote adaptive antitumor immunity. The pivotal role of the immune system in cancer treatment, in general, is highlighted by many clinical trials and success stories on using checkpoint immunotherapy. Hence, the potential of plasma treatment to induce ICD in tumor cells to promote immunity targeting cancer lesions systemically is also discussed.

## 1. Introduction

Cold physical plasma is a partially ionized gas operated at or around body temperature [1], and the term “plasma” in this work relates to this gas plasma and not to the protein-rich liquid of blood plasma. Physical plasmas are multicomponent systems as several plasma properties are described, including electrons and ions, electric fields, mild thermal and UV radiation, and reactive oxygen and nitrogen species. The latter will be abbreviated as ROS hereafter as most RNS also contain oxygen. It was recently outlined that ROS are major biomedical effectors of physical plasma treatment in biology and medicine [2].

Physical plasma is produced by different types of plasma devices such as the plasma jet [3–8], dielectric barrier discharge (DBD) [9–13], floating-electrode dielectric barrier discharge (FE-DBD) [14, 15], atmospheric pressure glow discharge torch (APGD-t) [16, 17], plasma brush [18], microhollow cathode discharge air plasma jet [19], microwave plasma torch [20], and nanosecond plasma gun [21]. Plasma jets and DBDs are particularly suitable for biomedical applications as these devices have already entered clinical practice [22]. The first report of using plasma in oncology was published in 2007 by showing the inactivation of melanoma cells *in vitro* following plasma treatment [14]. After that, more studies provided evidence of the anticancer capacity of plasma in several cancer types such as the brain [23–25], skin [26–29], breast [30–34], colorectal [35–37], lung [38–40], cervical [41–43], leukemia [44–48], pancreatic [49–54], liver [55–57], and head and neck [58–60]. Because of altered metabolism and mitochondrial dysfunction, cancer cells are often found to produce more intracellular ROS than nonmalignant cells [61–63]. In some studies, enhanced intracellular ROS in cancer cells makes them more susceptible to cell death induced by extracellular ROS [64, 65]. Among the extracellular ROS generated via plasma are superoxide anion, hydrogen peroxide, peroxynitrite, nitrite, nitrate, hydroxyl radicals, atomic oxygen, ozone, and singlet delta oxygen [66]. One hypothesis is that aquaporin transporters [67, 68] and lower levels of cholesterol in the membrane of cancer cells compared to a nonmalignant cell [69] increase the permeation of ROS through the cancer cell membrane, presumably via lipid peroxidation [70, 71]. As a result, more plasma-produced ROS are being transported to cancer cells, ultimately augmenting cell death. Cell death is a consequence of intracellular signaling regulated by pathways such as signal transducer and activator of transcription 3 (STAT3), MAP-kinase (MAPK) [72], and phosphatidylinositol 3-kinases (PI3K) via AKT (protein kinase B) [73]. Thus, plasma treatment can selectively target cancer cells because of their unique properties [67, 69].

Moreover, several pharmacological and physics approaches have been combined with plasma treatment to additively or synergistically augment toxicity in cancer cells. This includes radiotherapy [74–76], pulsed electric fields [77, 78], hyperthermia [79], photodynamic therapy [80], established anticancer drugs [81–85], and novel anticancer compounds [86, 87] including nanoparticles and emulsions [88–103]. This current will not focus on these aspects due to the broad nature of the combination approaches. Instead, plasma treatment has been described to directly or indirectly affect the immune system's cells, which may be harnessed in antitumor therapy [104], and current concepts are described that address this framework. Altogether, this review is aimed at unfolding the mechanisms, pathways, and immune-related activities involved in plasma cancer therapy.

## 2. Plasma Devices in Cancer Treatment

**Figure 1:**
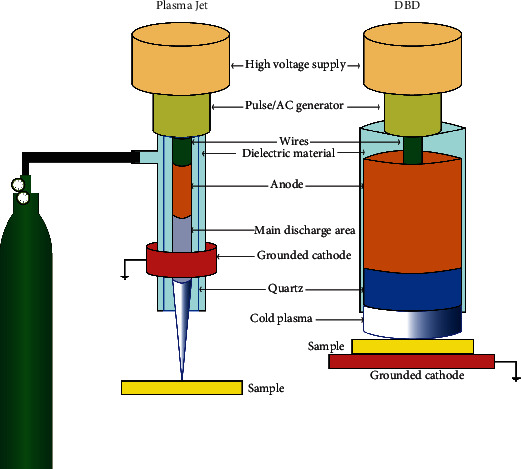
Schematic of the principles of plasma jets and dielectric barrier discharges (DBD). In plasma jets, the gas flow is required for the generation of cold physical plasma, while the plasma provided by DBD is created in ambient air. Plasma jets are grounded, while many DBD systems use the treatment target as a grounded cathode to produce cold physical plasma. Many types of gases can be used. Usually, noble gases such as argon, helium, and neon are employed, but air ionization is also feasible with specific parameter setups.

Analyzing plasma devices from different perspectives, including assessing their safety aspects, the capacity of ROS production, and cellular response to oxidative eustress and distress, are critical steps in examining how plasma works in cancer treatment. For a detailed overview of plasma physics, the reader is referred to reviews on this topic [105–107], while this section intends to give a brief overview to the biomedical audience. There are three major types of plasma sources including (1) DBD plasmas, also called “direct” plasma sources, that use the human body as an electrode; (2) plasma needles/plasma jets, also called “indirect” plasma sources, producing a discharge between two electrodes (Figure 1); and (3) hybrid plasma source, the combination of both (1) and (2) plasma sources [108]. The use of these plasma-producing systems depends on the study's purpose, and plasma jets seem more common than DBD plasmas based on several reports [109]. DBD plasmas' advantage is that they do not require a particular gas flow as needed in plasma jets, in which usually a noble gas is excited using high-frequency electrodes. In most cases with DBDs, the DBD electrode needs to be close to the target, and its diameter varies from several millimeters to centimeters [110]. Unlike DBDs, plasma jets use three common gases, including helium (He), argon (Ar), and nitrogen (N_2_), that determine the efficiency and pattern of ROS production [111]. Other critical parameters in operating plasma jets are, for instance, gas flow rate, applied voltage, and the distance from the nozzle to the target. For example, increasing the distance from the target to a jet nozzle decreases the concentration and variety of most reactive species reaching that target, while some, like ozone, are often found to be increased [112]. Also, changes in the feed gas flux are accompanied by changes in the reactive species composition, especially when the flow switches from laminar to turbulent [113, 114]. Another critical factor is exposure time. It is well known from microbiology studies that the growth inhibition zones of bacteria grown on agar increase with a rise in plasma treatment time [115]. Likewise, the plasma treatment time dictates the extent of biological responses, e.g., apoptosis [110, 116]. Also, exposure time can modulate the secretion of cell-signaling molecules, such as growth factors and cytokines [117]. The plasma devices' operating conditions affect the type and amount of reactive species products, especially in cancer cells [6, 118, 119]. Therefore, regulating these conditions improves plasma efficiency in inhibiting cancer cells (Table 1).

**Figure 2:**
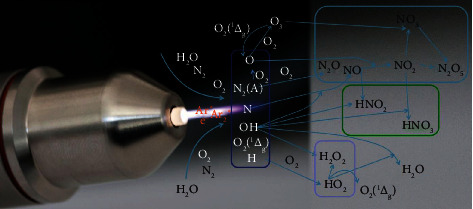
The atmospheric pressure argon plasma jet kINPen. The kINPen is a certified medical product in Europe and is regularly employed in dermatology. First initial trials in human cancer patients have been employed. Reproduced from [125].

One device that has been successfully employed in cancer treatment in patients [120, 121] is the atmospheric pressure argon plasma jet kINPen MED (Figure 2). Generally, medical plasmas are multicomponent systems, while it has been established that the biological activity of plasma treatment is mainly mediated via ROS/RNS and subsequent redox signaling [2, 122]. The ROS/RNS generation process is briefly described. For plasma jets, a gas is fed into the device. Usually, noble gases such as argon, helium, and neon are used to be ionized easily. These ionized gases are subsequently expelled into the ambient air. Reaction with oxygen and nitrogen takes place, generating reactive oxygen and nitrogen species. Within the plasma plume, hundreds of chemical reactions occur [123, 124], leading to the simultaneous generation of a large variety of reactive species [125]. These species have varying concentrations along the axis of the plasma jet and are characterized by individual travel distance and deterioration kinetics in the ambient air [126–128]. This aspect complicates the identification of exact species types and concentrations being delivered to the biological target. As a general measure of species quantification, liquids can be exposed to plasma to identify some species such as nitric oxide, singlet oxygen, hydrogen peroxide, hydroxyl radical, nitrite, nitrate, peroxynitrite, and ozone, based on established redox chemistry assays [66, 129–133]. Notably, there is no tool available to simultaneously investigate all types of species being generated in the plasma gas phase or treated liquids. This also holds true for plasma-treated tissues, as tools for assessing such species directly in such context are currently not available [134].

## 3. Cell Death Signaling in Cancer Cells

**Figure 3:**
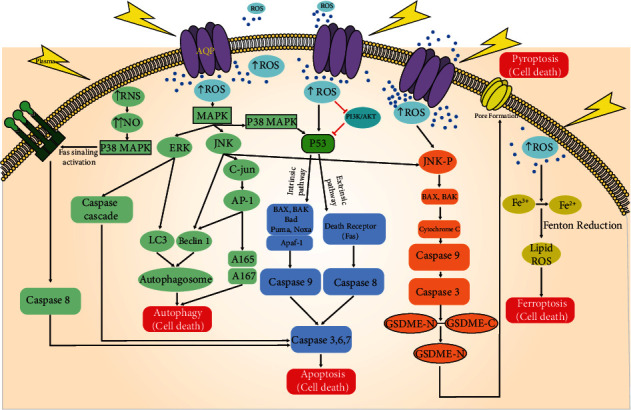
Model of three cell death signaling pathways in plasma-treated cancer cells. Plasma exposure increases aquaporin transporters in cancer cell membranes that allow the transport of H_2_O_2_ into the cells. Additionally, plasma treatment oxidizes cellular membranes, leading to cell death signaling. The excessive intracellular ROS contribute to the initiation of the cell death signaling (e.g., apoptosis, autophagy, pyroptosis, and ferroptosis) in cancer cells, partially through the activation of the MAPK pathway.

Several cell death modalities are known, including necrosis, apoptosis, necroptosis, autophagy, and pyroptosis [135]. These pathways can be a therapeutic target for the control and destruction of cancer cells. Deregulation of cell death signaling is a distinctive feature of cancer cells, and many cancer therapies target the apoptosis signaling machinery, including cell death receptors and mitochondrial signaling pathways [136, 137]. Direct plasma treatment or plasma-treated liquid (PTL) increases the intracellular ROS affecting different factors in cell death signaling in tumor cells [138]. Accordingly, targeting proteins or kinases involved in cell death signaling can efficiently induce apoptosis and death in the cancer cell. In the following, it will be outlined which pathways are involved in plasma-mediated cell death (Figure 3).

### 3.1. Apoptosis

It has been found that the ROS produced by plasma can induce cell death in cancer cells by activation of four MAPK pathways, including ERK1/2, c-Jun N-terminal kinase (JNK), p38 MAPK, and ERK5. The JNK and p38 pathways have a crucial role in the induction of apoptosis and the stress of the cells [139]. The activation of these pathways involves proapoptosis Bcl-2 proteins such as BAX and BAK that initiate intrinsic or mitochondrial cell death signaling. Also, p38 MAPK and JNK can upregulate p53 activity that controls cancer growth and triggers cell death in cancer cells [140].

p53 is a tumor suppressor protein involved in stress responses and intrinsic and extrinsic apoptosis pathways [141]. The phosphorylation of p53 triggers the intrinsic apoptosis pathway by activating the BH3 domain of proapoptotic proteins such as Puma, Noxa, Bad, Bax, Bak, and apoptosis-execution factors such as Apaf1 [136]. Also, p53 can initiate extrinsic apoptotic pathway signaling through cell death receptors such as Fas. Following activation of one of the two (or both) pathways, downstream caspases are activated, and apoptosis occurs. High levels of p53 protein in plasma-treated leukemia cells confirm p53-induced apoptosis [46, 136]. Moreover, increased Bax, Bcl-2, and caspase 8 expression in cancer cells after plasma treatment showed the effect of plasma on intrinsic and extrinsic pathways [46].

The ERK pathway is another MAPK pathway that coordinately regulates some essential biological functions of the cells, such as proliferation, differentiation, cycle regulation, apoptosis, and tissue formation. Also, this pathway can be related to tumor proliferation and invasion/metastasis [139]. Accordingly, the regulation of this pathway can be essential in inhibiting tumor cell growth. Plasma therapy has been shown to dramatically increase ERK1/2 phosphorylation and activate caspases in cancer cells, leading to their death [142].

Elevated intracellular ROS activates tumor suppressor proteins and kinases, suppressing the oncogenic PI3K/AKT pathway. Thus, inhibition of the PI3K/AKT pathway initiates cancer cell death [140]. PI3K/AKT signaling mediates a wide range of cellular functions, including transcription, translation, proliferation, growth, and survival. This pathway maintains the balance between cell proliferation and apoptosis in cancer cells and is associated with metastasis in some tumors [143]. Also, PI3K/AKT activation has been shown to play a critical role in inhibiting p53. Indeed, plasma therapy downregulates the PI3K/AKT pathway and induces p53-mediated apoptosis and cancer cell death [97]. Nitric oxide (NO), a product of NOX activity in some tumor and innate immune cells, has pro- and anticancer effects. Depending on its intracellular level, low NO levels can promote tumor cell growth, while high NO levels usually cause the tumor cell to die [144]. Physical plasma treatment enhances intracellular NO levels in cancer cells, leading to MAPK p38 activation [144]. This plasma-derived NO was shown to significantly increase the presence of active caspases 3 and 8, confirming the role of plasma in activating caspase cascade and inducing cell death [144].

Moreover, activating protein-1 (AP-1) as a dimeric transcription factor, Fra-1, and c-Jun (highly expressed in invasive cancers) enhance cancer cells' migration and proliferation. Their phosphorylation is often regulated by MAP kinases such as JNK and p38 [145]. Plasma treatment can modulate the expression of AP-1 related transcription factors in cancer cells such as leukemia. It has been reported that JUND, a subfamily of Jun, can trigger phagocyte activation and cytokine secretion such as IL-8 in plasma-treated THP-1 cells [146].

Another pathway involved in cancer cell death is signal transducer and activator of transcription 3 (STAT3) signaling. STAT3 has a role in proliferation, survival, migration, invasion, and angiogenesis [147]. Therefore, targeting this pathway can be efficient in cancer cell inhibition. Plasma-treated osteosarcoma showed an initiation in the apoptotic pathway by reducing phosphorylation in the AMPK or STAT3 pathways, which had an inhibitory effect on cancer cells' growth [148]. Further experiments are needed to explain the effect of plasma on the STAT3 pathway in this area.

### 3.2. Autophagy

Autophagy is a process that occurs in all cells to eliminate dysfunctional or damaged cell organelles. The autophagic process plays a double-edged sword role in cancer progression [149]. Regulation of autophagy is mediated by tumor suppressor proteins such as LC3 and Beclin-1, leading to cancer cells' death. Various environmental stressors such as starvation, hypoxia, and growth factor deprivation can convert LC3 to LC3-II by conjugating a lipid molecule called phosphatidylethanolamine (PE) to incorporate into the autophagosome membrane. Also, Beclin-1 is involved in the very early stage of autophagosome formation [149, 150]. Plasma-produced ROS increased autophagosome formation through activate ERK1/2 and induce LC3. This is presumably due to ROS stimulating the JNK pathway to phosphorylate Bcl-2 and releasing Beclin-1 associated with LC3 involved in autophagic cell death [151, 152]. Using PTL decreases the phosphorylated mTOR and AKT protein levels, which is critical for cancer cell viability. Besides, PTL increases LC3B expression in endometrial cancer cells. So PTL can inhibit cell viability while inducing autophagic cell death in endometrial cancer cells [153]. Moreover, PTL treatment increases the level of LC3A/B, p-ERK kinase, which is involved in Beclin-1-related autophagy. Indeed PTL induces apoptosis of pancreatic cancer cells through the ROS-dependent autophagy pathway [154]. As a result, JNK phosphorylates the c-Jun protein, which leads to the production of AP-1, which in turn promotes the expression of many genes such as Bax and FasL [151].

### 3.3. Pyroptosis

Pyroptosis is another type of programmed cell death mediated by the gasdermin family which includes GSDMA, GSDMB, GSDMC, and DFNA5/GSDME [155]. Pyroptosis has some characteristics of apoptosis as well as necrosis. Pyroptotic cells undergo nuclear condensation and chromatin DNA fragmentation, similar to apoptotic cells. In parallel, cell membrane pore formation, cell swelling, cell membrane rupture, and the release of proinflammatory mediators, including IL-1*β*, IL-18, ATP, and HMGB14 during pyroptosis, occur, sharing similar features to necrosis. Therefore, pyroptosis is an inflammatory form of cell death and has a bilateral role in tumor cell progression. Plasma treatment was shown to induce pyroptosis in cancer cells via ROS, promoting the phosphorylation of JNK and increasing cytoplasmic cytochrome C levels [156]. These pathways induced caspase 9 and caspase 3 activation by cleaving GSDME, which induces pyroptosis in cancer cells [157].

### 3.4. Ferroptosis

Ferroptosis is an iron-dependent and reactive oxygen species- (ROS-) reliant cell death distinct from apoptosis, classic necrosis, autophagy, and other forms of cell death at morphologic, biochemical, and genetic levels [158–160]. Ferroptosis is mainly based on cytological changes, including decreased mitochondrial cristae, a ruptured outer mitochondrial membrane, and a condensed mitochondrial membrane. Excessive membrane lipid peroxidation and the occurrence of oxidative stress cause cell abnormalities in ferroptosis [160]. This form of cell death can be induced by small molecules such as erastin and Ras-selective lethal small molecules (RSL). Also, iron and ROS accumulation, activation of the MAPK pathway, and release of arachidonic acid mediators trigger this type of cell death. However, xCT (SLC7A11) and glutathione peroxidase 4 (GPX4) are critical regulators of ferroptosis [159]. In addition, p53 might act as a rheostat, preventing ferroptosis under basal or low ROS stress while promoting ferroptosis in high oxidative stress conditions [161]. p53 represses the expression of SLC7A11, a vital component of the cystine/glutamate antiporter. Hence, p53 can inhibit cystine uptake and sensitizes cells to ferroptosis [162]. Moreover, another study implicated that plasma treatment increases cell death in the samples with lower xCT expression than samples with higher xCT expression [163].

Excess irons are the basis for ferroptosis. Interestingly, redox-active iron pools (i.e., Fe_2_^+^) via Fenton reaction can directly catalyze lipid peroxides, which cause ferroptosis [162]. Accordingly, it was hypothesized that plasma exposure could induce destruction of the shell of ferritin and simultaneous reduction from Fe(III) to Fe(II), resulting in Fenton reaction to cause oxidative cell death [164]. Also, plasma exposure may kill oral squamous carcinoma cells through ferroptosis, dependent on ample catalytic Fe(II) [165]. Further studies are required to demonstrate the effect of plasma therapy in cancer cell ferroptosis.

## 4. Immune Cell Activation Followed by Plasma Treatment

Plasma treatment can affect the activation of immune cells and their ability to provide effective antitumor immunity [166]. As currently known, antitumor immune responses consist of innate and adaptive immunity that interacts and acts on cancer cells by various means [104]. The innate immune system can both foster and limit cancer progression through direct interaction with tumor cells and the activation of other cells in the tumor microenvironment (TME) [167, 168].

### 4.1. Immunogenic Cancer Cell Death (ICD)

Induction of cell death is an expected valuable outcome in plasma-treated cancer cells. It may also cause tumor cells to externalize or secrete many types of damage-associated molecular patterns (DAMPs), including ATP, high mobility group protein B1 (HMGB1), calreticulin (CRT), and heat shock protein 90 (HSP90), leading to the recruitment of immune cells [169]. CRT and ATP are critical for innate immune cell activation to uptake dead tumor cells to occur in the inflammatory context. This mediates an antitumor immune response by promoting DC maturation and antigen presentation, resulting in T-cell responses against tumor cells [170].

**Figure 4:**
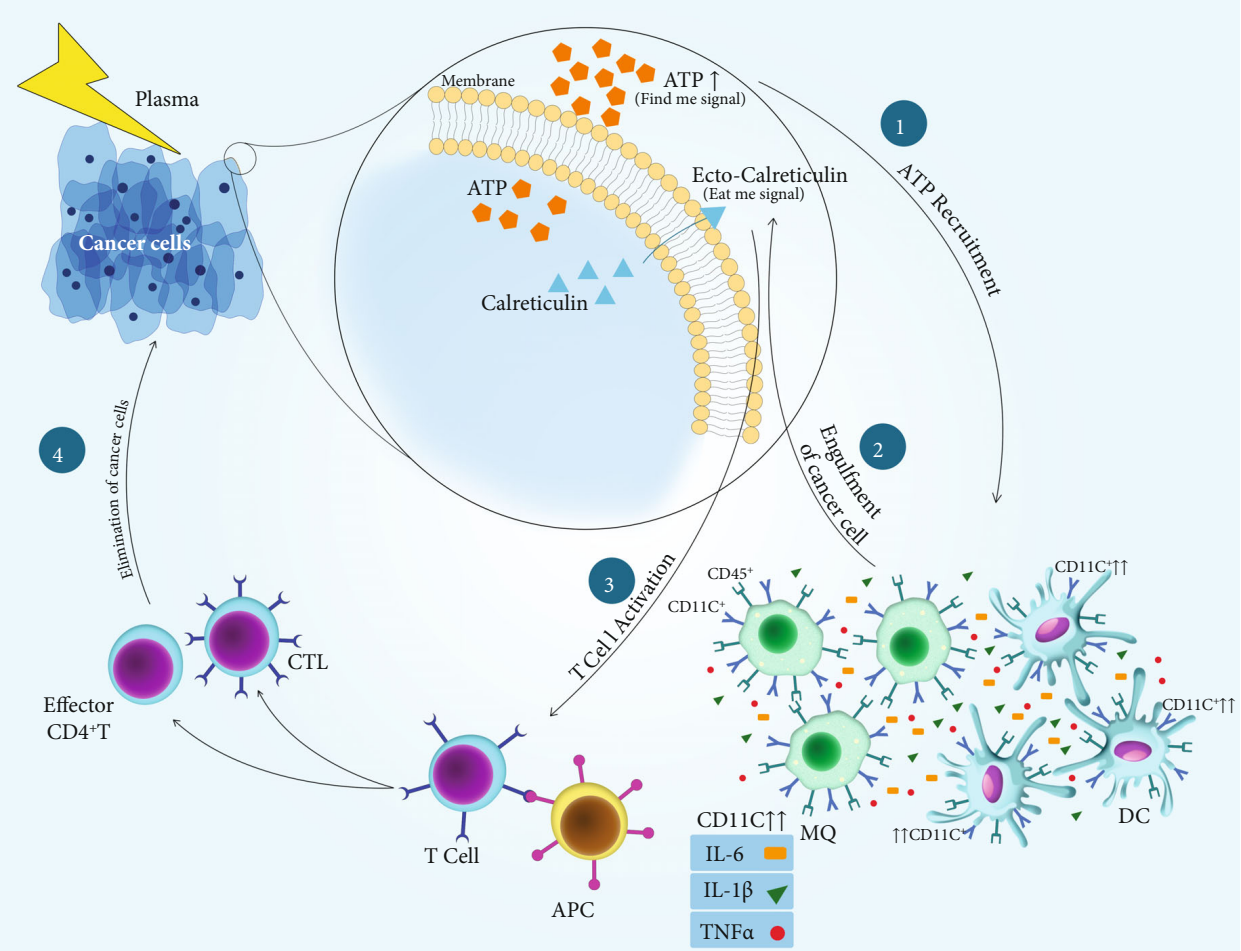
Model of plasma-induced immunogenic cell death in cancer cells. Plasma exposure leads to an increase in DAMP signaling (e.g., ATP and calreticulin), which (1) provides inflammatory stimuli for (2) promoting the processing of cancer cells by APCs. Consequently, (3) activated APCs promote the development and activation of (4) effector T-cells, capable of precisely and systemically eradicating cancer cells distant from the site of plasma treatment.

It has been demonstrated that nonthermal plasma treatment induces ICD by the generation of ROS [171] and other charged species [166] and increases the immunogenicity of tumor cells. Plasma upregulates immunogenic cell surface molecules such as MHC-I [172] and surface-exposed calreticulin (ecto-CRT). The latter acts as an *Eat Me* signal facilitating the recognition, engulfment, and processing of tumor cells by APCs. High levels of extracellular ATP following plasma therapy [173] act as a *Find Me* signal for the recruitment and activation of APCs in tumor microenvironments (Figure 4). Increased expression of CD45, a leukocyte marker, and CD11c, an APC marker, in the tumor microenvironment of BALB/c mice exposed to the plasma suggested additional leukocytes' recruitment, including APCs, presumably via DAMP signaling [174, 175].

### 4.2. Macrophages

Macrophages are critical immune cells in the TME and play a pivotal role in immune homeostasis. In response to a wide variety of environmental conditions, macrophages can differentiate and polarize into different phenotypes of M1 and M2. Tumor cells release and express molecules that hijack macrophages, supporting tumor growth [176]. In some cancer types, such as in the pancreas and brain, up to 50% of the cells are macrophages, continually supporting angiogenesis and phagocytose, silently and without inflammation, dead tumor cells. These are called tumor-associated (M2) macrophages. M2 macrophages express CD163 (scavenger receptor) and CD206 (mannose receptor) as anti-inflammatory markers and arginase. In addition, they release IL-10, TGF-*β*, and PGE2 and have a higher expression of PD-L1 that can repress antitumor T-cell responses. In turn, however, macrophages can also be licensed to kill tumor cells in the presence of proper proinflammatory stimuli, called proinflammatory (M1) macrophages [167]. M1 macrophages, as classically activated macrophages, express CD68, CD80, and CD86 costimulatory molecules and can control tumor progression by releasing TNF-*α*, IL-1*β*, IL-12, and iNOS. In the appropriate setting, some cytokines such as INF-*γ* can convert M2 macrophages to the M1 phenotype in the TME [177, 178]. Another study, however, found an M2 skewing of monocyte-derived macrophages with plasma treatment [179]. While plasma-treated monocytes generated ROS and were susceptible to plasma-induced cell death, as shown before [180], plasma-treated macrophages were not [178].

Using human monocytes, plasma treatment was shown to exacerbate M1 macrophages' cytotoxic activity against tumor cells. This was accompanied by an increased expression of CD86 (M1 marker) and low levels of CD163 and CD206 (M2 markers) on the THP-1-derived macrophages [178]. A similar increase of toxicity was made in A549 lung cancer cells during coculture with THP-1-derived macrophage *in vitro* [181]. Another study reported that the rate of cell death in a plasma-treated nasopharyngeal carcinoma cell line (CNE-1) cocultured with native (M0) macrophages (macrophages) was higher than the presence of macrophages, possibly due to the increase in extracellular ATP [182]. In such coculture systems of cell-line-derived macrophages and tumor cells, elevated levels of TNF-*α* were also linked to the increased cytotoxicity observed [183]. TNF-*α* inhibits the tumor progression by activating CD8^+^ T-cells and induces inflammatory cytokines such as IL-1, IL-6, IL-8, and cytotoxic factors like NO and ROS produced by macrophages and NK cells [184, 185]. Strikingly, recent evidence suggests that plasma treatment supports monocytes' differentiation process into macrophage-like cells. In contrast to the other studies, this was found in cell lines and using primary monocytes isolated from the human blood [186]. Moreover, plasma treatment of cancer cells and culturing monocytes in these DAMP-containing cancer cell supernatants promoted monocyte activation [123] and their cytotoxicity upon coculture with tumor cells [187].

### 4.3. Cross-Talk between Dendritic Cells and T-Cells

Activation of T-cells and the generation of long-lived memory cells in the tumor microenvironment (TME) are the critical target of cancer therapies. CD8^+^ T-cells are the key player in the adaptive immune system for the direct killing of cancer cells via the release of cytotoxins, such as perforin and granzyme B. Effector CD4^+^ T-cells in response to an antigenic tumor can secrete cytokines such as IFN-*γ*, TNF-*α*, and IL-2 that limit tumor progression and help the activation of CD8^+^ CTL in a later stage [188]. Activation of adaptive T-cell responses depends on antigen recognition, so antigen-presenting cells (APC) such as DCs play a critical role in stimulating an adaptive immune response, especially cytotoxic CD8^+^ T-cells and CD4^+^ T-cells. DCs are innate immune cells known as professional APC and play a crucial role in linking innate and adaptive immune responses. DCs phagocytose, process, and present the tumor antigens to naïve antigen-specific CD4^+^or CD8^+^ T-cells via major histocompatibility complexes (MHC) II and I, respectively. There are two major subsets of DC: classical/conventional DC (cDC) and plasmacytoid DC (pDC). pDC produces type I interferons, which are essential in the stimulation of antitumor immune response. They can also generate regulatory T-cells (T_reg_) in the tumor microenvironment, which favors tumor progression. Depending on their subtype, cDCs present tumor antigens to prime both CD8^+^ and CD4^+^ T-cells [189].

It was previously speculated that plasma-derived ROS treatment of tumor cells initiates the cancer-immunity cycle by promoting ICD, DC maturation, and priming of antitumor T-cells in the draining lymph node [190, 191]. A recent *in vivo* report supports this claim by providing evidence for ICD and subsequent DC activation together with checkpoint therapy-augmented plasma and abscopal effects in a melanoma model [192]. Using the same cell type but a different type of plasma source, ICD and the subsequent protection from tumor growth in a preventive vaccination model were shown, and mechanistically, the effects were deduced to the action of short-lived ROS [193]. *In vitro*, plasma-treated PBS activity on tumor cells may be involved in DC maturation. Also, higher levels of TNF-*α* and IFN-*γ* and decreased levels of immunosuppressive cytokines such as TGF-*β* produced by DC cocultured with tumor cells exposed to plasma-treated PBS an immune-enhancing effect of this approach [194]. Moreover, other in vitro studies suggested a distinct cytokine profile and modest but evident DC activation in the presence of directly plasma-killed tumor cells [74, 173]. It was recently reported for a translational research-relevant plasma jet accredited as a medical device in Europe that plasma treatment not only induced ICD in melanoma cells that were successfully used as a preventive vaccine in mice but also was accompanied by an increased influx of CD4^+^ and CD8^+^ T-cells in the TME along with their increased activation and memory phenotype [195]. Moreover, increased efficacy of plasma treatment was demonstrated when combined with a toll-like-receptor (TLR) agonist activating DCs and superior efficacy of one plasma-derived ROS cocktail rich in atomic oxygen over other ROS cocktails. These findings corroborated previous reports on increased T-cell infiltrates in plasma-treated syngeneic melanomas *in vivo* [196, 197]. In addition, it was recently shown in vitro and in vivo that the immunogenicity plasma-treated protein can confer immunoprotection in mice against melanoma growth [198], giving rise to entire novel concepts in plasma oncotherapy [199].

## 5. Clinical Trials and Case Series on Plasma Therapy in Medicine including Cancer

*In vivo* and *in vitro* studies in plasma medicine have shown promising results, encouraging clinicians to evaluate plasma therapy in clinical settings across several types of diseases (Table 2). Since there is only a few reports on plasma anticancer studies, other clinical applications are described as well in the following. For each plasma device, such studies must demonstrate safe plasma treatment in the first clinical step.

### 5.1. Case Series

Several case series and reports have reported on the use of different plasma devices in humans to treat disease. In Greifswald, Germany, clinicians investigated the clinical application of cold physical plasma treatment in 21 patients with advanced head and neck cancer in a palliative setting. This study was aimed at evaluating tumor surface changes and the ratio of apoptotic cancer cells, respectively, in group I and group II. Among the 12 patients in group I, there was no enhanced or stimulated tumor growth under two weeks after cold physical plasma treatment. The result of 9 patients in group II showed more frequent apoptotic cells in tissue areas treated by plasma than in untreated areas [120, 121, 200].

Moreover, German clinicians used a plasma device to treat six patients suffering from wound healing disturbances after maxillofacial surgical procedures. The size and localization of the defect were different among all cases, so plasma therapy was initiated at various postsurgery times, ranging from 2 to 42 weeks. The primary outcome showed complete healing, defined as wound closure and the absence of any signs of infection. Besides, the secondary outcomes showed complete remission after 48 weeks of plasma treatment. In that study, several therapeutic properties of plasma, including antibacterial effect, stimulation of tissue repair, regeneration, neovascularization, and skin microcirculation, were considered. Based on the results, plasma is a promising approach to treat chronic healing disorders of wounds resulting from CMF surgery [201]. Another study by the same authors evaluated the effect of plasma therapy on wound healing disorder following the radial forearm free flap (RFFF) procedure. The endpoint of this therapy showed the successful remission of wounds. It was concluded that plasma treatment possibly is a new therapeutic modality to avoid repeated surgery [202].

Actinic keratosis (AK) was another skin disease that has been investigated to be treated using plasma. In one study, 17 lesions were plasma-treated and followed up for one month without interval evaluation. Three lesions improved significantly, and the condition of five lesions did not worsen. Interestingly, none of the patients experienced side effects, such as pain and inflammation during treatment [203]. All patients showed a decline in AK characteristics such as erythema, scaling, crusts, and thickness, and in some cases, the total lesion number was decreased [204].

The efficacy of plasma therapy has also been investigated in the treatment of wart lesions. The results of one study revealed that all lesions of the first patient faded after 2 to 3 plasma exposure cycles. In a second patient, however, the lesions were improved but did not disappear completely [205]. The same authors demonstrated that plasma exposure could also be an effective modality for wart treatment in pediatric patients [206]. The plasma device used in this study is currently not approved by the FDA. Nevertheless, plasma treatment was suggested to induce apoptosis in malignant cells *ex vivo* [207], so it seems likely that this also holds for premalignant cells. However, clinical data are insufficient to confirm the plasma mechanism in improving the wart [206].

Overall, plasma therapy is a novel promising therapeutic tool in managing tumor cells and the recovery of infection, postoperative wound healing, actinic keratosis, and wart disorders.

### 5.2. Clinical Trials

The first registered clinical trial on plasma cancer treatment was initiated in Tübingen, Germany, in 2017 to manage cervical intraepithelial neoplasia. Approximately 170 patients were planned to participate in the study. Final results, however, were not reported yet apart from the observation that pathological remission and HPV reduction were secondary outcomes [208]. In 2019, a U.S. company used plasma to treat 20 patients with breast and lung cancer after standard treatments, including chemotherapy, radiation, and surgery. Preliminary results suggested a preferential targeting of tumor cells, but further confirmation is awaited. This technology was the first to be approved in an FDA phase I clinical trial in August 2019 [209]. Moreover, the Skin Center Dermatology Group investigated the effect of plasma to treat 100 subjects with skin disorders. This study enrolled 100 participants suffering from actinic keratosis, acne, or verruca plana. The results of the plasma treatment were successful in most cases and showed no side effects. However, this study has not yet been completed and final results are awaited [210]. A recent clinical study examined the effect of plasma in the treatment of hair loss. This study started on June 8, 2020, and is currently recruiting. However, no results have yet been reported [211].

## 6. Side Effects of Plasma Treatment

Any medical treatment has to meet both efficacy and safety requirements. While many studies had investigated the efficacy of plasma treatment in many types of diseases, studies on their safe applications are less frequent. The main agents of biomedical plasma effects, ROS, are also produced during physiological processes in the body. Hence, ROS are not toxic or dangerous *per se*, but their exacerbated concentration or application frequency might be. To understand this from a practical point of view, it needs to be mentioned that H_2_O_2_ at molar concentrations (e.g., 3% equals 1M) is used for wound disinfection and dentistry. For comparison, to reach the concentration of 1M H_2_O_2_ in 1 ml of a saline solution, this would translate to a plasma treatment time of 30.000 min (or 500 h) for an accredited argon plasma jet [212]. For *in vitro*, *in vivo* (mice), and patient treatment with this jet, typical treatment times are between 5 s and 3 min [213, 214]. This calculation emphasizes that the ROS doses generated with plasma treatment range from inducing ROS-related (cell death) signaling rather than overloading the cells with necrotic doses of ROS and would account for most medically suited plasma devices currently in use.

Nevertheless, several safety studies have been performed, especially for the well-characterized kINPen MED [215]. This plasma jet does not generate mutagenic events, as shown using the OECD-accredited HRPT test and the cytokinesis-block micronucleus assay [216, 217]. Notably, the phosphorylation of the histone 2A-X seems a secondary event due to plasma-induced cell death rather than direct DNA damage [218]. *In vivo*, no formation of micronuclei was observed [219]. In a wound-healing model in mice, the animals were plasma-treated for seven days using the kINPen, and one year later, the animals were investigated using MR-imaging, CT-scanning, histopathology, and tumor marker analysis in the blood and tissues [220]. No tumor formation or any other detrimental long-term effect was observed. Concerning mucosal tissue in mice, plasma exposure caused mild inflammation, and the epithelial layers healed without showing signs of hyperplasia or dysplasia [221]. Side effects in patients were recently summarized [215] and currently extended to the first 5-year follow-up in plasma-treated wounds [222]. In patients suffering from advanced squamous cell carcinoma of the head and neck, some side effects such as bad taste, fatigue, and bleeding were seen in some cases after plasma treatment. However, all of the side effects were mild to moderate and not life-threatening [223].

For plasma devices other than the kINPen MED, safety has been implied as well, albeit less systemically. For instance, this accounts for the PlasmaDerm and SteriPlas devices [224–227], and the efficacy and safety of plasma wound treatment have been reviewed in a meta-analysis recently [228]. Moreover, the number of clinical trials indicates a preevaluation (e.g., CE mark in Europe) of the safety across many other plasma sources as a prerequisite to clinical use. In Europe, several medical plasma device types have been employed over the last seven years in over 100 clinical centers and thousands of applications already without any note of severe side effects. It is important to note that there are many plasma devices for cosmetic application on several international markets, but their safety has been addressed to a minimal extent only in most cases. Besides, several plasma devices or device modifications that are not in clinical use yet but are aimed for such application have undergone in vitro or in vivo risk assessments already [229–234]. A DIN spec has been published in Germany that suggests several assays that should be performed for a standard characterization of medical plasma devices [235]. A DIN spec is a legal norm in Germany that describes detailed methods and assays to characterize a product, process, or device based on industry consensus. Current efforts are aimed at generating a respective ISO-norm for the safety of plasma devices that would harmonize risk assessments to ensure the safety and efficacy of plasma treatment of human diseases.

## 7. Conclusion

This review summarizes the recent advances in understanding plasma therapy in medicine, emphasizing cancer treatment. Studies of plasma therapy in the clinical setting have only begun, but promising results were reported so far. Cold physical plasma alters many features of tumor cells, ultimately leading to their demise. Plasma also promotes inflammatory signaling pathways that can augment antitumor responses by innate and adaptive immune cells. Further studies are required to demonstrate the effect of plasma on memory cells' generation against tumor cells. Because plasma releases tumor-associated antigens and facilitates antigen processing, using a combination of plasma and immunotherapy regimens, such as immune checkpoint inhibitors, possibly enhances antitumor immune responses.

## Figures and Tables

**Table 1:** Plasma devices and their characteristics and outcomes in tumor cells.

Plasma device	Gas/modality	ROS/RNS investigated	Species/cell or tissue type	Biological consequence			Ref.
				Proliferation	Apoptosis	Migration	
Plasma jet	He/direct	O	Human (G361 melanoma)	↓	n.i.	↓	[4]
Plasma jet	He, O_2_/direct	O and OH	Human (HCT-116, SW480 colorectal carcinoma)	↓	↑	↓	[35]
Plasma jet	Ar/indirect	OH, singlet oxygen radicals, NO_2_	Human (NOS2, NOS3 epithelial ovarian carcinoma)	↓	↑	n.i.	[236]
Plasma jet	He, O_2_/direct	O and OH	Human (BHP10-3 and TPC1 thyroid papillary carcinoma cell lines)	=	=	↓	[73]
Plasma jet	Ar/indirect	H_2_O_2_	Human (SK-Mel-147 melanoma cell line)	=	=	-	[237]
Plasma jet	He/indirect	OH	Human (RPMI8226 and LP-1 MM cell line)	↓	↑	↓	[238]
Plasma jet	Ar/indirect	H_2_O_2_	Human (HEC-1 and GCIY endometrial and gastric cancer)	↓	n.i.	n.i.	[239]
Plasma jet	Air/indirect	H_2_O_2_	Human (ES2, SKOV3, and WI-38 cell lines)	=	↑	↓	[240]
Plasma jet	Air/direct and indirect	OH	Human (U87 MG brain cancer cells)	↓	↑	n.i.	[241]
DBD plasma	Air/direct	-	Human melanoma cell line (ATCC A2058)	n.i.	↑	n.i.	[14]
DBD plasma	Air/direct	O_3_, NO, HO_2_, H_2_O_2_, OH, O	Human (MCF10A breast cancer)	=	↑	n.i.	[12]
DBD plasma	Air/direct and indirect	O_2_^−^, H_2_O_2_	Human (U87MG glioblastoma) and Human (HCT-116 colorectal carcinoma)	↓	↑	n.i.	[15]
DBD plasma	Air/direct	-	Human (T98G brain cancer cell line)	↓	↑	n.i.	[11]
DBD Plasma	Air/direct	-	Human (T98G malignant)	↓	n.i.	n.i.	[13]
DBD plasma	Air/direct	H_2_O_2_ and NOx	Human (H460 lung cancer cell lines)	n.i.	↑	↓	[10]
DBD plasma	Air/direct	H_2_O_2_, O_3_, OH	Human (A549 lung adenocarcinoma epithelial cells)	↓	↑	↓	[110]
DBD plasma	Air/indirect (plasma-treated macrophages)	N_2_	Human (U251MG and U87MG cells) cocultured with plasma-treated macrophages	↓	↑	↓	[178]

DBD: dielectric barrier discharge; HPMCs: human primary mesothelial cells; HEC-1: human endometrial carcinoma; PTL: plasma-treated liquid; ROS: reactive oxygen species; Ar: argon; He: helium; O_2_: oxygen; N_2_: nitrogen; NOx: nitric oxides; NO: nitric oxide; H_2_O_2_: hydrogen peroxide; HO_2_: hyperoxide; O_3_: ozone; O_2_^−^: superoxide; O: atomic oxygen; OH: hydroxyl radicals; NO_2_: nitric dioxide; n.i.: not investigated.

**Table 2:** Clinical case series and trials on plasma therapy in medicine.

Study type	Year	Condition	# of pat.	Plasma source/certification	Outcome with plasma treatment	Allocation	Ref.
Case series	2015	Infected wounds	11	kINPen MED (certified)	Complete healing of the wounds	N/A	[242]
Case series	2016	Advanced head and neck cancer	21	kINPen MED (certified)	No enhanced tumor growth and more apoptotic cell kill	N/A	[121]
Case report	2016	Percutaneous driveline infection	1	kINPen MED (certified)	Completed regression of local infection	N/A	[243]
Case series	2016	Nonhealing wounds	4	kINPen MED (certified)	Completed wound repair	N/A	[202]
Case series	2017	Nonhealing wounds	6	PlasmaDerm (certified)	Completed remission	N/A	[201]
Case series	2017	Actinic keratosis	5	FE-DBD (not certified)	17 lesions: 9 showed full regression, 3 significantly improved, 5 showed no change	N/A	[203]
Case series	2018	Warts	2	FE-DBD (not certified)	Patient 1: wart cleared; patient 2: wart improved but not cleared	N/A	[205]
Case series	2018	Therapy-resistant corneal infections	4	kINPen MED (certified)	Significantly elimination of pathogens	N/A	[244]
Case series	2018	Actinic keratosis	7	SteriPlas (certified)	Overall decline of actinic keratosis characteristics	N/A	[204]
Case series	2020	Warts	5	FE-DBD (not certified)	4 warts cleared, 1 did not change	N/A	[206]
Clinical trial	2011-2012	Chronic venous leg ulcers	14	PlasmaDerm (certified)	Significantly improved ulcer size-reduction	Random	[245]
Clinical trial	2016-2020	Actinic keratosis, acne, verruca plana	100	Plasma to treat skin lesions and acne	Successful cure in most of them	Nonrandom	[210]
Clinical trial	2017-2020	Facial wrinkles, rhytides	55	J-Plasma He-jet (FDA approved)	Significant improvement, no serious adverse events	N/A	[246]
Clinical trial	2017-2020	Cervical intraepithelial neoplasia	170	Plasma treatment	Pathological remission and HPV reduction	Nonrandom	[208]
Clinical trial	2017-2018	Intact skin, experimental contaminant added to patient skin	25	Plasma	Safety, efficacy, and efficiency of plasma for burn wound treatment	Nonrandom	[247]
Clinical trial	2017-2019	Onychomycosis of toenail	5	Plasma treatment	Mycological cure, evident nail growth	N/A	[248]
Clinical trial	2019	Wound healing	100	Cold argon Plasma	Ongoing, no results have been yet reported	Random	[249]
Clinical trial	2020	Androgenetic alopecia	40	Plasma-treated aqueous-alcohol solution	Ongoing, no results have been yet reported	N/A	[211]
